# Momentum informed muon scattering tomography for monitoring spent nuclear fuels in dry storage cask

**DOI:** 10.1038/s41598-024-57105-y

**Published:** 2024-03-20

**Authors:** JungHyun Bae, Rose Montgomery, Stylianos Chatzidakis

**Affiliations:** 1 Oak Ridge National Laboratory, Oak Ridge, TN 37830 USA; 2 School of Nuclear Engineering, Purdue University, West Lafayette, IN 47907 USA

**Keywords:** Cosmic ray muon, Muon scattering tomography, Nondestructive examination, Dry storage cask, Spent nuclear fuel, Nuclear fuel, Nuclear waste

## Abstract

Development of an effective monitoring method for spent nuclear fuel (SNF) in a dry storage cask (DSC) is important to meet the increasing demand for dry storage investigations. The DSC investigation should provide information about the quantity of stored SNF, and quality assurance of materials should be possible without opening the cask. However, traditional nondestructive examination (NDE) methods such as x-rays are difficult to deploy for DSC investigation because a typical DSC is intentionally designed to shield against radiation. To address this challenge, cosmic ray muons (CRMs) are used as an alternative NDE radiation probe because they can easily penetrate an entire DSC system; however, a wide application of muons is often hindered due to the naturally low CRM flux (~10^4^ muons/m^2^/min). This paper introduces a newly proposed imaging algorithm, *momentum-informed muon scattering tomography (MMST)*, and presents how a limitation of the current muon scattering tomography technique has been addressed by measuring muon momentum. To demonstrate its functionality, a commercial DSC with 24 pressurized light water reactor fuel assemblies (FAs) and the MMST system were designed in GEANT4. Three noticeable improvements were observed for MMST system as a DSC investigation tool: (1) a signal stabilization, (2) an enhanced capability to differentiate various materials, and (3) statistically increased precision to identify and locate missing FAs. The results show that MMST improves the investigation accuracy from 79 to 98% when one FA is missing and 51% to 88% when one-half FA is missing. The advancement of the NDE technique using CRM for DSC verification is expected to resolve long-standing problems in increasing demand for DSC inspections and nuclear security.

## Introduction

Spent nuclear fuels (SNFs) are continuously generated in the nuclear reactor core, and one-third is replaced by fresh fuels every 18 or 24 months to maintain reactor efficiency^1^. Discharged SNF assemblies are immediately transferred to a storage pool (often referred to as *wet storage*) adjacent to the reactor to cool down decay heat and decrease radiation emission intensities. SNF assemblies are stored in a storage pool for years before they are moved to on-site dry storage casks (DSCs), which are designed to remove residual decay heat using natural circulation of air and shield the radiation using metal canisters and concrete overpacks^2,3^. Because it is not allowed to open the DSCs once they are sealed, monitoring of the material integrity and accountancy of SNF assemblies inside the DSC are required without opening it. A nondestructive examination (NDE) approach to DSC monitoring with typical radiographic probes is highly challenging because DSCs are designed to shield radiation and prevent radiation exposure to the environment. For example, steel canister walls (sometimes with lead plates) shield gammas, and concrete overpacks with borate plastic materials absorb neutrons^4–6^. Although x-rays and ultrasonic tomography techniques have been devised for the NDE, their applications are limited to local investigation such as monitoring of defects of cask materials and their sealing with weld seam^7–9^. In addition, a fast neutron interrogation tomography was developed for the NDE method for monitoring SNF in the casks. However, neutron scanning must be performed around the canister without concrete shielding^10–12^.

Cosmic ray muons (CRMs) are considered a promising nontraditional radiation probe for the NDE method of DSC monitoring because they easily penetrate the entire DSC without absorption whereas they change their flight direction with measurable angles because of consecutive Coulomb scatterings with nuclei and electrons. These unique features of CRMs enabled scientists to devise a new imaging technique, muon scattering tomography (MST)^13–15^. For decades, CRMs have been widely used for many engineering applications including monitoring SNF assemblies in DSCs^16–24^. Although the results are promising, the applications of muon tomography are often limited because of CRMs’ wide energy spectrum and angular distribution. Therefore, without measuring the energy and incoming direction of CRMs, possibilities would be limited with respect to improving muon tomography image resolution. To measure muon energy in the field for muon tomography applications, a few technical approaches have been developed by using time-of-flight^25,26^, scattering angle analysis^27,28^, and Cherenkov radiation^29,30^. In this work, we use a Cherenkov muon spectrometry’s energy measurement capability because it is field-deployable, technically simple, and easily coupled with muon detectors^31–33^.

Based on the measurement capability of scattering angle and energy analysis offered by muon detectors and spectrometries, we developed a new imaging algorithm that includes muon energy information in the existing MST imaging algorithm, called *momentum-informed muon scattering tomography (MMST)*. Both MST and MMST use a point-of-closest-approach algorithm to locate scattering positions^34,35^. However, MST uses scattering angles for density mapping, whereas MMST uses *M-values*, which encode momentum and scattering angle parameters in a single variable. The MMST algorithm does not increase computational costs because an *M-value* is an independent variable in the objective function that simply replaces an old variable. To demonstrate the benefits of the new imaging algorithm, a DSC with 24 pressurized light water reactor (PWR) SNF assemblies was modeled, and CRMs were generated using a high-energy particle transport code, GEANT4^36,37^. Then, the computational simulation results of muon interactions with the DSC and SNFs were analyzed using MATLAB. Visual and statistical inspections with MST and MMST algorithms are performed for surveying the DSC when, one-half, one, and two fuel assemblies (FAs) are missing out of 24 FAs.

The objectives of this work are to (1) demonstrate the advantages of measuring muon energy in MST applications, (2) verify the functionality of the newly developed imaging algorithm, MMST, and (3) discuss the improvement of statistical reliability and image resolution when MMST is used for DSC investigations. The overall image quality is improved by adapting MMST algorithm and it enables to visually locate one and one-half missing fuel assemblies in the DSC. From the signal analysis results of scattering angles and *M-values*, three improvements were observed: (1) scattering angle and *M-value* signals are stabilized, (2) density mapping of structural materials and SNFs becomes distinctive, and (3) the detection capability of finding missing FAs in DSC is improved. The statistical signal analysis results show that the MMST can find and locate a missing FA with a 98% confidence level (CL) compared with 79% for MST. Additionally, the MMST algorithm successfully locates a position of a missing one-half FA with an 88% CL, which was challenging with MST because of its low CL (51%).

## Cosmic ray muon

Except for neutrinos and protons, muons are the most abundant cosmic particles at sea level. Cosmic ray muons are known as secondary cosmic particles because they are produced by pion and kaon decays during extensive air showers caused by interactions between high-energy primary cosmic particle (i.e., protons, electrons, and heavy ions such as O^+^ and N^+^) and air molecules in the Earth's atmosphere^38^. Muons are similar to electrons, but they are approximately 207 times heavier and decay to electrons and neutrinos with a mean lifetime of 2.2 μs^39^. Although the best estimate of CRM flux at sea level is approximately 70 m^−2^ s^−1^ sr^−1^, it varies depending on various geometrical factors such as zenithal angle, azimuthal angle, altitude, longitude, latitude, and solar activities^38^.

### Cosmic ray muon energy spectrum

**Figure 1:**
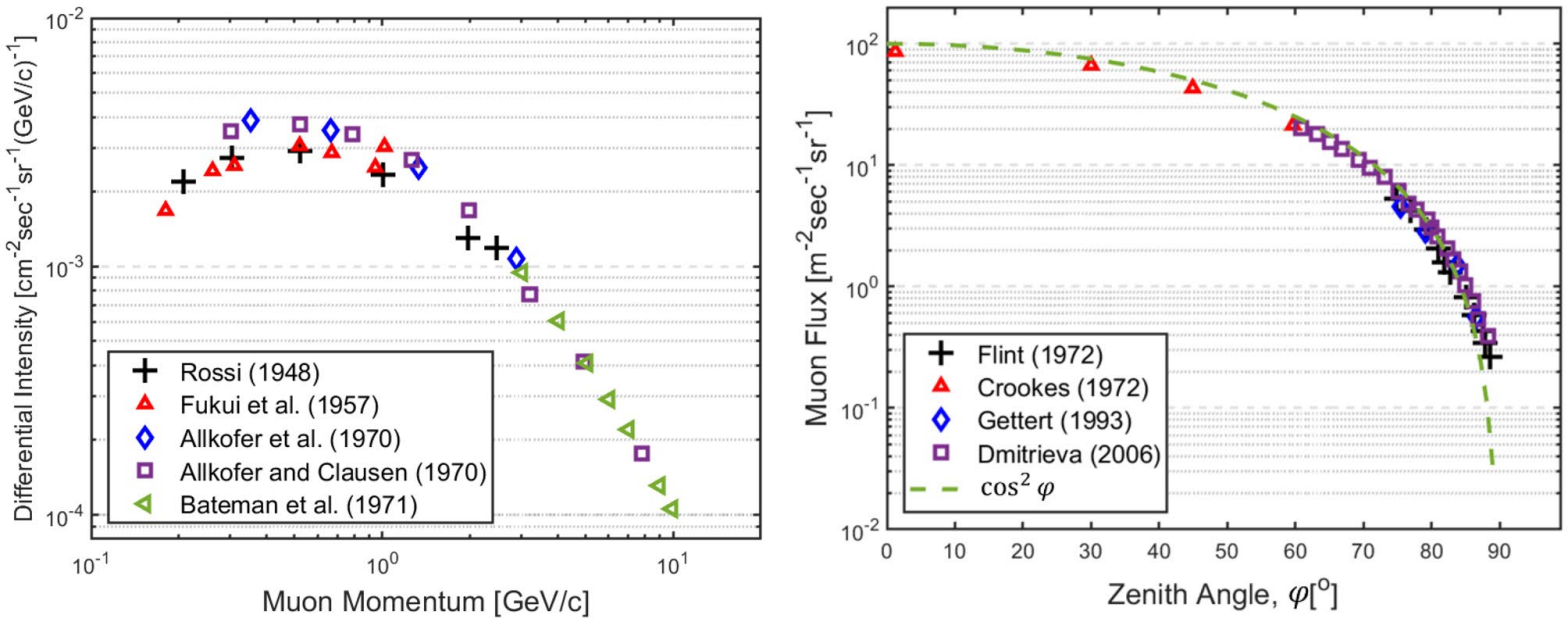
Differential intensity of cosmic ray muons in the range of 0.1–10 GeV/c and the variation of muon flux with a zenith angle range of 0°–89° at sea level^54^.

The muon energy spectrum was derived by exploiting three phenomena—production spectra from kaons and pions, energy loss in the atmosphere, and decay. The differential CRM energy spectrum at sea level is given by^38^:

1 $$\frac{d{N}_{\mu }}{d{E}_{\mu }\mathrm{d\Omega }}\left[{{\text{cm}}}^{-2}{{\text{s}}}^{-1}{{\text{sr}}}^{-1}{{\text{GeV}}}^{-1}\right]\approx 0.14{E}_{\mu }^{-2.7}\left[\frac{1}{1+\frac{1.1{E}_{\mu }{\text{cos}}\varphi }{115 {\text{GeV}}}}+\frac{0.054}{1+\frac{1.1{E}_{\mu }{\text{cos}}\varphi }{850 {\text{GeV}}}}\right],$$

where *dN*_*μ*_*/dE*_*μ*_*dΩ* is the expected muon counts with respect to muon energy per solid angle, and *φ* is the muon zenith angle. The first and second terms on the right-hand side represent contributions of pions and kaons, respectively. The experiment results of CRM measurements in the muon momentum range of 0.1–10 GeV/c at *φ* = 0° are shown in Fig. 1.

### Cosmic ray muon angular distribution

The angular distribution is another important CRM profile with the energy spectrum because it varies depending on the incoming flight direction of muons or the zenith angle. A cosine-squared model is widely used because of its simpleness and accuracy.

2 $$I\left(\varphi \right)={I}_{0}{{\text{cos}}}^{2}\varphi ,$$

where *I*_*0*_ is the vertical muon flux (*φ* = 0°). A new approximation model was developed to address two limitations of the cosine-squared model, (1) a non-zero muon flux at *φ* = *9*0° problem and (2) a point detector assumption. Therefore, a muon zenith angle distribution can be written as

3 $$\frac{d{N}_{\mu }}{d\varphi }=\left(\frac{2\pi }{3}\right)\left(1-{\text{cos}}\gamma \right)\left[{\left({\text{cos}}\left(\varphi -\gamma \right)+{\text{cos}}\left(\varphi +\gamma \right)\right)}^{2}-{\text{cos}}\left(\varphi -\gamma \right){\text{cos}}\left(\varphi +\gamma \right)\right],$$

where

4 $$\gamma =\frac{1}{2L}\left[{\text{ln}}\frac{4{L}^{2}+1}{{\left({L}^{2}+1\right)}^{2}}+\frac{1}{L}{{\text{tan}}}^{-1}2L+2\left(L-\frac{1}{L}\right){{\text{tan}}}^{-1}L\right],$$

and

5 $$L\equiv \frac{{r}_{d}}{D} ,$$

where *φ* is the zenith angle, and *γ* is defined as the projection angle. Here, *γ* accounts for a 3D angular range that detectors can measure, and it is determined by the distance, *D*, and radius of detector, *r*_*d*_^40^. Zenith angle experimental measurements of CRMs in the range of 0–89° at sea level are shown in Fig. 1.

## Muon scattering tomography

When a muon interacts with matter, it loses energy and is deflected mainly due to the inelastic collisions with electrons and consecutive collisions with nuclei, or multiple Coulomb scattering (MCS)^38^. The muon deflection angle due to a single Coulomb scattering is described using the Rutherford formula:

6 $$\frac{d\sigma }{d\Omega }={\left(\frac{z{e}^{2}}{4\pi {\epsilon }_{0}}\frac{1}{4{E}_{k}}\right)}^{2}\frac{1}{{{\text{sin}}}^{4}(\theta /2)} ,$$

where *dσ/dΩ* is the scattering cross section into a unit solid angle, or differential scattering cross section, *θ* is the muon scattering angle, *z* is the atomic number of a target, *e* is the electron charge, *ϵ*_*0*_ is the permittivity of the vacuum, and *E*_*k*_ is the initial kinetic energy for the projectile (muon) in MeV. Unless a target object is extremely thin, a muon will undergo MCS and the sum of multiple random deflection angles can be approximated with a Gaussian distribution^41^. The number of scattered muons between *θ* to *θ* + *dθ*, can be approximated using a 2D radial Gaussian distribution, which is given by

7 $$\frac{dN}{d\theta }=\frac{\theta }{\sqrt{2\pi }{\sigma }_{\theta }}{e}^{-{\theta }^{2}/2{\sigma }_{\theta }^{2}} ,$$

where *θ* is the muon scattering angle, and *σ*_*θ*_ is the standard deviation. *σ*_*θ*_ can be estimated by material properties and muon momentum, and it is given by^42^

8 $${\sigma }_{\theta }=\frac{13.6 {\text{MeV}}}{\beta cp}\sqrt{\frac{X}{{X}_{0}}}\left[1+0.038{\text{ln}}\left(\frac{X}{{X}_{0}}\right)\right] ,$$

where *X* is the thickness of material, *X*_*0*_ is the radiation length, *p* is the muon momentum, and *βc* is the muon velocity in terms of the speed of light, *c*. Radiation length, *X*_*0*_, is a nuclear property, and it can be found in the Particle Data Group library^43,44^. For composite and unconventional material, the *X*_*0*_ value can be approximated within < 1% error by the following analytical formula^45^:

9 $${X}_{0}\left[{\text{g}}/{{\text{cm}}}^{3}\right]=\frac{716.4A}{z\left(z+1\right){\text{ln}}\left(\frac{287}{\sqrt{z}}\right)} ,$$

where *A* is the atomic number.

### Position mapping method

**Figure 2:**
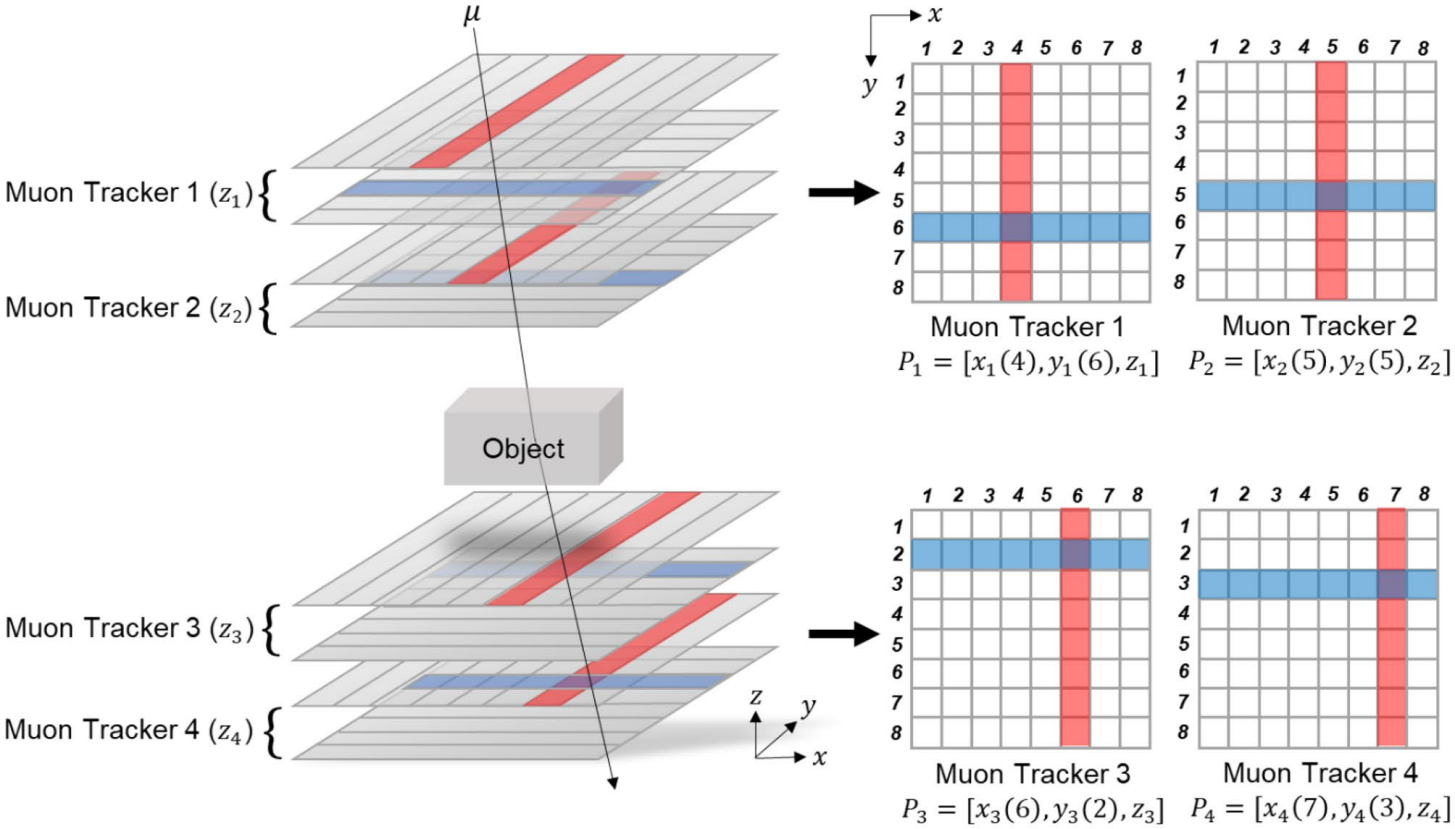
Reconstruction of incoming and outgoing muon trajectories using two-fold upper and lower muon trackers. Four 8 by 8 muon trackers are shown in the example.

**Figure 3:**
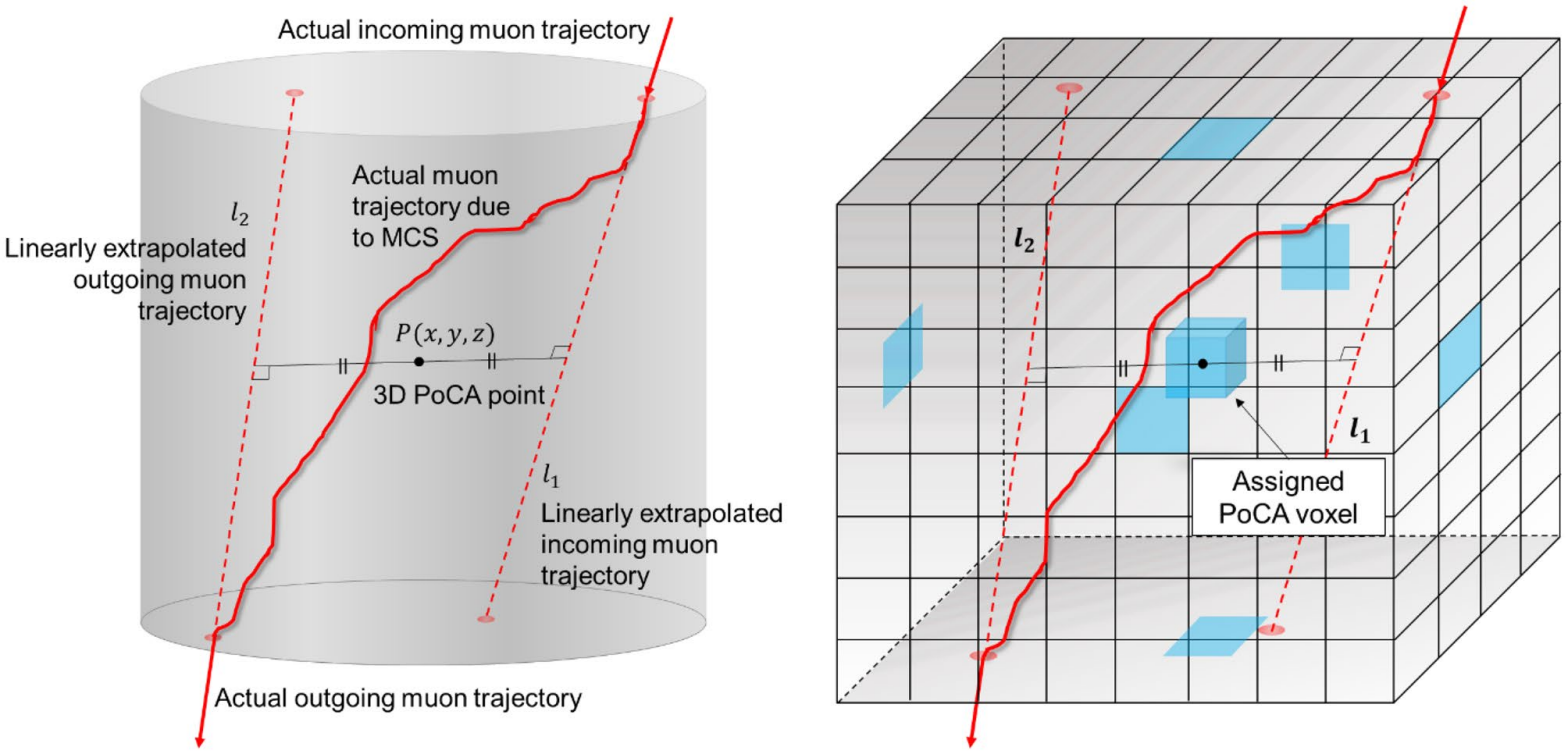
Visualized PoCA algorithm to locate a single scattering point using incoming and outgoing muon trajectories (left) and the assigned PoCA voxel in a 3D voxelated volume of interest (right).

To reconstruct images of objects using MST, muon scatterings must be located for position mapping. However, it is almost impossible to reconstruct the actual scattering trajectory of muons in a medium because the measured scattering angle is a result of consecutive random small deflections, although some studies successfully reduce uncertainty in the muon trajectory estimation using Bayesian analysis^46^ and maximum likelihood expectation maximization^47^. To address this limitation, a simple and fast algorithm, a point-of-closest Approach (PoCA), was developed to efficiently locate a single scattering position^34^. However, there is a key assumption in the PoCA algorithm: a muon’s trajectory changes once in the medium. To find the PoCA point, we need to measure incoming and outgoing muon trajectories. The incoming muon trajectory, or initial muon trajectory before interacting with target objects, is reconstructed using two muon trackers, as shown in Fig. 2. The outgoing muon trajectory, the final muon trajectory after interacting with target objects, is also reconstructed in the same manner. Both incoming and outgoing lines are extrapolated until the shortest distance between two lines in 3D is achieved, and the middle point of a distance between two lines is a PoCA point in 3D. Then, a PoCA voxel is assigned, which includes the PoCA point in a voxelated volume of interest, as shown in Fig. 3.

From the measurement in upper and lower muon trackers, the 3D coordinates of a muon are

10 $${P}_{{\text{upper}}}={\left(x,y,z\right)}_{1 \,{\text{or}}\, 2} ,$$

11 $${P}_{{\text{lower}}}={\left(x,y,z\right)}_{3 \,{\text{or}}\, 4} ,$$

where (*x, y, z*)_*i*_ is the 3D muon coordinate at the *i*th muon tracker. The extrapolated lines, *l*_*1*_ and *l*_*2*_, using two muon coordinates at the upper and lower muon trackers are

12 $${l}_{1}={\overrightarrow{k}}_{1}\cdot {\overrightarrow{e}}_{1},$$

13 $${l}_{2}={\overrightarrow{k}}_{2}\cdot {\overrightarrow{e}}_{2},$$

where ***e***_***1***_ and ***e***_***2***_ are the unit directional vectors for incoming and outgoing muon trajectories. ***K***_***1***_ and ***k***_***2***_ are the coefficient vectors to extend lines toward the region of interest. Then, the 3D PoCA point is given by

14 $$P\left(x,y,z\right)=\frac{1}{2}{\text{min}}\Vert {l}_{1},{ l}_{2}\Vert .$$

This process is repeated for every recorded muon event, and PoCA voxels can contain multiple PoCA points.

### Density mapping method

To complete images of objects using MST, the assigned PoCA voxels must be visually distinguished using colors or a density mapping. The assigned PoCA voxels are colored based on the muon scattering angle, which is an angular difference between *l*_*1*_ and *l*_*2*_ from Eqs. (12) and (13). The scattering angle, *θ*_*s*_*,* is given by

15 $${\theta }_{s}=\sqrt{\Delta {\theta }_{x}^{2}+\Delta {\theta }_{y}^{2}} ,$$

where *Δθ*_*x*_ and *Δθ*_*y*_ are the angle difference between *l*_*1*_ and *l*_*2*_ in terms of x- or y-axis. If there exist multiple PoCA points with different scattering angles in a PoCA voxel, the averaged scattering angle is used.

16 $${\overline{\theta }}_{s}=\frac{1}{{N}_{v}}\sum_{{N}_{v}}{\theta }_{s,n},$$

where *N*_*v*_ is the total number of recorded muon events in the PoCA voxel.

## Modeling

### Cosmic ray muon simulations

**Figure 4:**
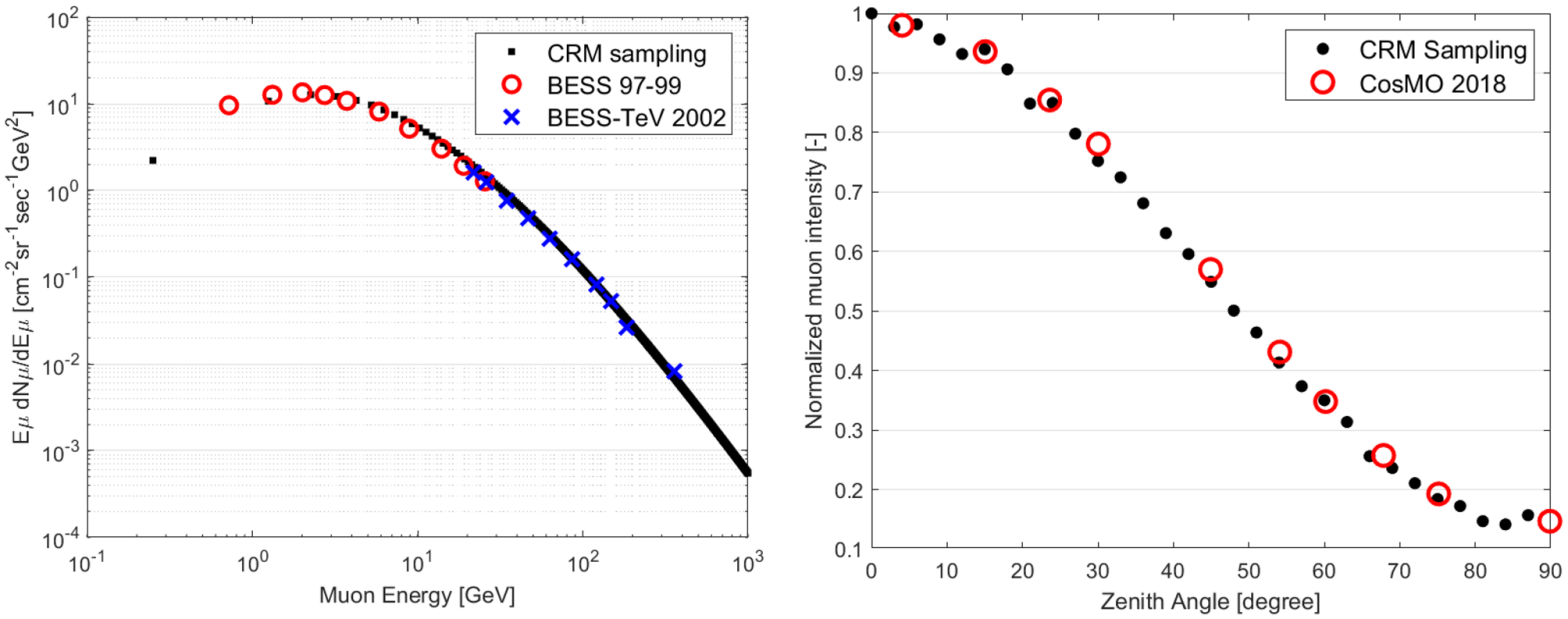
Muon energy spectrum and zenith angle distribution from the CRM sampler and experimentally measured data from BESS 97-99^48^, BESS-TeV 2002^49^, and CosMO^50^.

To provide statistically reliable CRM samples for muon tomography and monitoring applications, a new CRM sampler was developed based on phenomenological models of muon energy spectra and angular distribution. The CRM sampler generates muon samples from the described energy spectrum (Eq. 1) and angle distribution (Eq. 3) with user-defined parameters—number of sample generations, maximum and minimum energy limits, and zenith angles, as well as their uncertainties. Because Eqs. (1) and (3) are not standard probability distributions, statistical algorithms are employed for generating random samples. The inverse transform method is used to obtain random samples based on probability profiles from both probability distribution functions, but the insignificant probabilities (< 5 × 10^−6^) are dropped. The generated CRM samples are saved in a table format so that it can be exploited in GEANT4. The examples of muon energy spectrum and zenith angle distribution from the CRM samplers are shown in Fig. 4.

### Spent nuclear fuel dry cask storage

**Figure 5:**
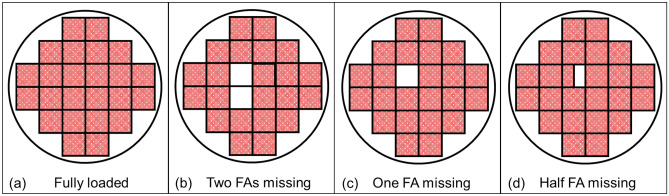
Layouts of SNF assemblies in a dry cask when (**a**) they are fully loaded and when (**b**) two, (**c**) one, and (**d**) one-half of middle FA(s) are missing.

SNFs are stored in various designs of storage depending on fuel type, capacity, usage, and manufacturer^51^. In general, the SNF dry cask consists of two major components: a stainless-steel canister and concrete overpack. The outermost surface of a dry cask is made of thick concrete (800–1000 mm) to shield radiation from SNF decay and is designed to maintain the total radiation dose equivalent rate at the site less than 0.25 mSv per year at the controlled boundary of the system^52^. In this work, we used a commercial SNF dry cask canister model that stores up to 24 PWR FAs. Each FA includes 15 × 15 UO_2_ fuel rods (*ρ*_UO2_ = 10.97 g/cm^3^), which have a radius, pitch, and length of 5.35, 14.3, and 3658 mm, respectively. The overall dimension of each FA is 215 mm × 215 mm × 3,658 mm. The array of FAs is surrounded by an annular concrete shielding (*ρ*_concrete_ = 2.3 g/cm^3^) with an inner and outer radius of 863.5 and 1685 mm. In simulations, we modeled 23½, 23, and 22 PWR FAs to represent one-half, one, or two middle FAs that are missing, as shown in Fig. 5.

### Muon detector and spectrometry

**Figure 6:**
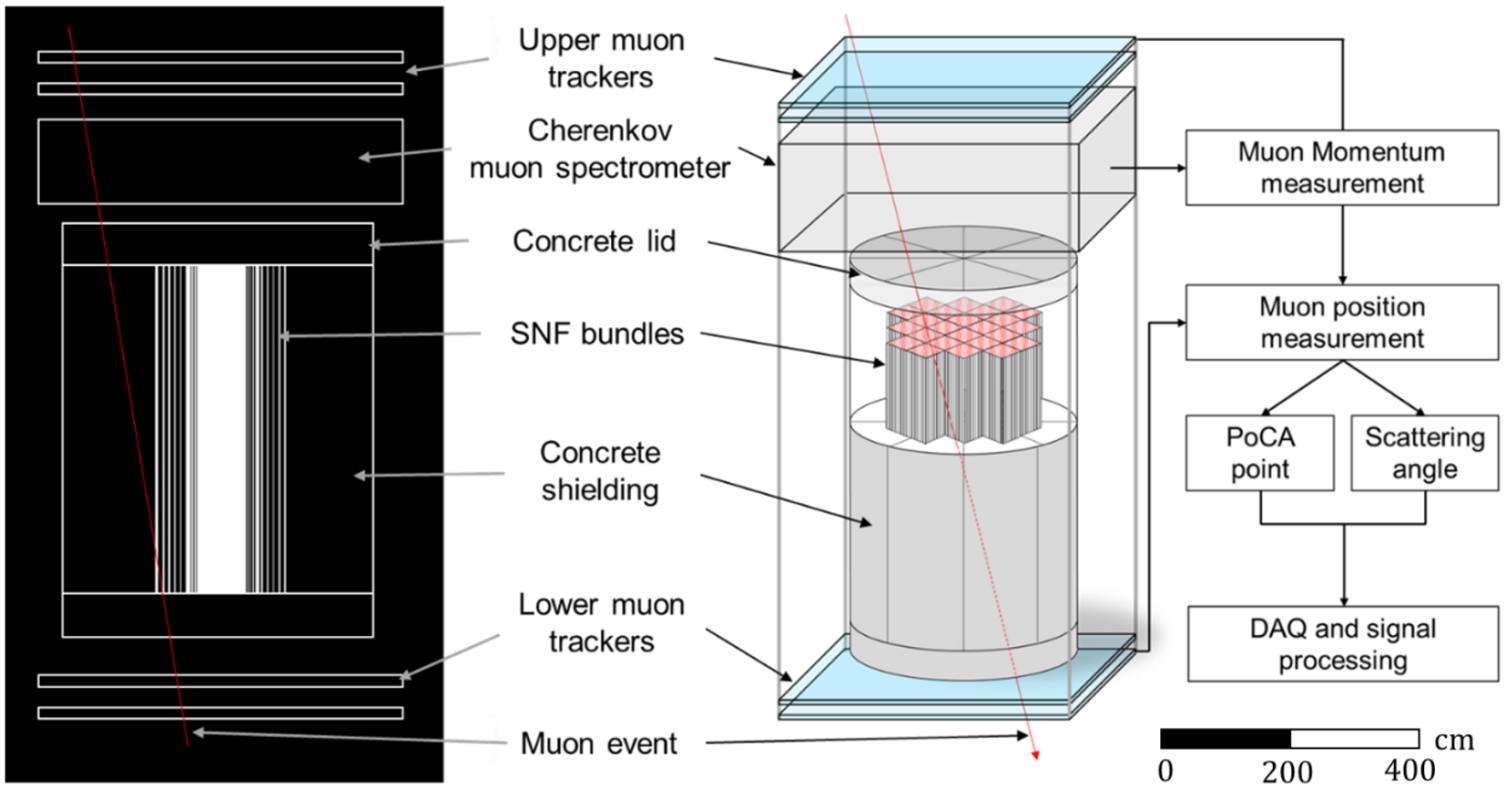
Overview of instrumentations and DSC for the momentum-informed muon scattering tomography system using a Cherenkov muon spectrometry for SNF imaging and monitoring.

To measure positions and momentum for each muon event during simulations, two muon trackers were designed and placed above and below the SNF DSC and Cherenkov muon spectrometry. The muon trackers consist of 4 m × 4 m upper and lower scintillators. The distance between upper and lower trackers is 6.0 m, and that of two scintillators is 0.3 m. A Cherenkov muon spectrometry was located 0.1 m below the upper tracker to measure the incoming muon momentum. The active area of the Cherenkov spectrometry is equivalent to the size of muon trackers (4 m × 4 m), and the overall length is 1.0 m. Moreover, the SNF DSC was placed between the muon spectrometry and lower tracker. The overall length and diameter of DSC are 3.66 m and 3.37 m, respectively. Figure 6 provides an overview of the implementation of the Cherenkov muon spectrometry in the muon tomography instrumentations around the SNF DSC and visualized GEANT4 simulations. In our simulations, 10^5^ and 10^6^ cosmic ray muon samples were generated for the SNF DSC imaging, which can be translated to a day and a week of scanning times in practice.

## Results and discussion

### Momentum-informed muon scattering tomography

After CRM samples are stochastically generated from the muon sampler, muons interact with various structures, including muon trackers, spectrometry, and SNF DSC. The output data from GEANT4 simulations provides posterior muon energy and positions from the installed Cherenkov muon spectrometry and muon trackers, respectively. Incoming and outgoing muon trajectories were reconstructed from output data, and the PoCA points were determined from them. Then, PoCA voxel and scattering angle computed using Eqs. (14) and (15) were saved in a vector. The 3D coordinates of PoCA voxel, *P*_*x,y,* and *z*_, and scattering angle, *θ*, for each muon event, or *muon event vector*, is written by

17 $$V_{i} |_{\text{MST}} = \left[ {P_{x} , P_{y} ,P_{z} ,\theta } \right]_{i}\, i = 1, \ldots , N_{\mu } ,$$

where *N*_*μ*_ is a total number of recorded muon events and the subscript MST represents that Eq. (14) is used for the MST algorithm. In a typical MST algorithm, tomographic images of target objects are reconstructed based on Eq. (17). In this work, a Cherenkov muon spectrometry was incorporated into the typical MST imaging system to include muon momentum information. Therefore, Eq. (17) can be augmented by adding an extra element, *p*, which is the recorded muon momentum in GeV/c.

18 $${V}_{i}|_{{\text{MMST}}}={\left[{P}_{x}, {P}_{y},{P}_{z},\theta ,p\right]}_{i}\, i=1, \dots , {N}_{\mu }.$$

To simultaneously consider two parameters, *θ* and *p* in Eq. (18), we developed a new algorithm encoding momentum and scattering angle behaviors into a single variable (*M-value*). An expression to compute *M-value* from *θ* and *p* is mathematically derived based on the exponentially inverse-proportional relationship between most probable muon scattering angle and momentum (details can be found in^53^).

19 $${\text{ln mod}}\left(\theta \right)=k{{\text{log}}}_{10}p+M,$$

where *k* and *M* are slope and y-intercept. Although *k* slightly varies from − 2.56 for heavy nuclei to − 2.23 for light nuclei, its variance is negligible and was considered as a constant, *k* =  − 2.4, for the SNF DSC simulations. On the other hand, *M* explicitly depends on types and sizes of materials, and it is a useful parameter to represent material properties. To calculate an *M* or *M-value* for each muon event, Eq. (19) was modified using *θ*, instead of using mod(*θ*). Thus, a new function, *M* (*p*, *θ*), is given by

20 $$M\left(p,\theta \right)\equiv {{\text{log}}}_{10}\left({\theta }^{2.3}/{p}^{k}\right),$$

when *k* is a constant, − 2.4, and Eq. (20) becomes

21 $$M\left(p,\theta \right)={{\text{log}}}_{10}{\left({\theta }^{0.958} [{\text{rad}}]\times p [{\text{GeV}}/{\text{c}}]\right)}^{2.4}.$$

In the MMST technique, *M-value* is facilitated to replace the scattering angle. Therefore, Eq. (18) can be updated and simplified by substituting *θ* and *p* with *M*,

22 $${V}_{i}|_{{\text{MMST}}}={\left[{P}_{x}, {P}_{y},{P}_{z},M\right]}_{i}\, i=1, \dots , {N}_{\mu }.$$

Therefore, PoCA voxels are mapped based on *M* density to reconstruct tomographic images.

### Monitoring spent nuclear fuel dry storage cask

**Figure 7:**
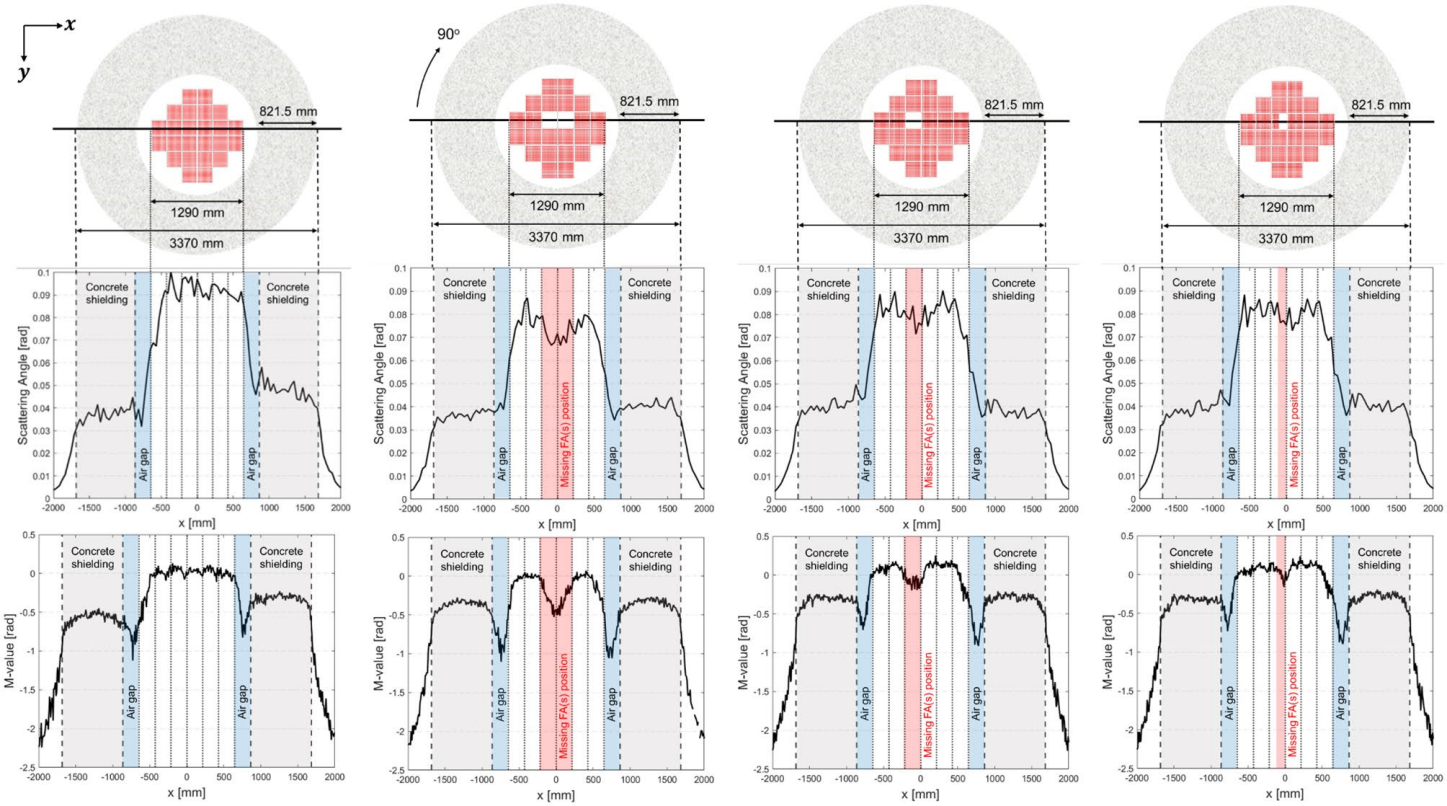
Layouts of SNF assemblies, concrete overpacks, and airgap in the DSC when FAs are fully loaded, two middle FAs are missing, one middle FA is missing, and one-half FA is missing (from left to right). Averaged scattering angle and *M-value* signal distributions (0 < y [mm] < 215) are plotted along the x-axis (− 2000 < x [mm] < 2000).

Two NDE technical approaches were used to monitor SNF in the DSC and its structure, (1) a signal analysis and (2) an image analysis. In the signal analysis approach, scattering angle and *M-value* distributions for the FAs and concrete overpacks were compared when 0, 1/2, 1, and 2 FAs are missing. The signal distributions were computed at the horizontal line, which crosses the surrounding air, concrete, airgap, and FAs as shown in Fig. 7. The signal amplitude distributions of scattering angles and *M-values* were averaged over 0–215 mm (size of one FA) in terms of y-axis, and the results are plotted along the x-axis from − 2000 to 2000 mm. Although both scattering angle and *M-value* signal amplitudes are theoretically proportional to the material density, scattering angle signal amplitudes of the airgap (38–50 mrad) were similar or greater than concrete overpacks in the MST signal analysis because of the strong interference signals around the airgap such as FAs (75–83 mrad). This high-uncertainty problem is resolved in the MMST signal analysis by showing that the airgap has the lowest *M-values* (− 0.80 to − 0.55) among other structural materials as shown in Fig. 7. The averaged signal amplitudes for scattering angles and *M-values* for various structural materials with different number of missing FAs in the DSC are summarized in Tables 1 and 2, respectively.

**Table 1:** Averaged scattering angle signal amplitudes in MST for various structural materials in the DSC and various numbers of missing FAs.

Number of missing FAs (out of 24 FAs)	Structural materials			
	FAs [rad]*	Missing FAs [rad]	Concrete [rad]	Airgap [rad]**
0	0.083	–	0.038	0.042
½	0.080	0.079	0.039	0.048
1	0.081	0.078	0.038	0.050
2	0.075	0.071	0.039	0.038

*Averaged signal amplitudes from 6 FAs in a row except missing FAs.

**Airgap between FAs and concrete overpacks which is highlighted in blue in Fig. 7.

**Table 2:** Averaged *M-value* signal amplitudes in MMST for various structural materials in the DSC and various numbers of missing FAs.

Number of missing FAs (out of 24 FAs)	Structural materials			
	FAs [–]	Missing FAs [–]	Concrete [–]	Airgap [–]
0	0.01	–	− 0.38	− 0.80
½	0.09	− 0.03	− 0.27	− 0.55
1	0.10	− 0.10	− 0.30	− 0.55
2	− 0.01	− 0.29	− 0.41	− 0.74

By comparing the signal distribution results from scattering angles and *M-values*, three improvements were observed, (1) signal stabilization, (2) clear distinction among different materials, and (3) enhanced capability to detect missing FAs. The signal stability, or reduced signal fluctuation, can be achieved because the uncertainty in scattering angles is significantly reduced by including a muon momentum term as described in Eq. (21). Similarly, a difference of signal amplitudes for different materials becomes distinctive by using *M-values* instead of scattering angles. In particular, the capability of capturing the airgap signals between FA bundles in the center and concrete overpacks (highlighted in blue in Fig. 7) is noteworthy because it implies that the MMST method can find the missing FAs as well as possible cavities in the DSC structure. Therefore, the overall capability to identify the number and exact location of missing FAs in the DSC is significantly improved by using *M-value* signal distribution profile. The averaged scattering angle and *M-value* signal amplitudes are changed from 75 to 71 mrad, and − 0.01 to − 0.29 where two missing FAs exist. The signal amplitude reduction ratios were 5.0% and 13%, respectively. Similarly, the averaged scattering angle and *M-value* signal amplitudes decreased from 80 to 79 mrad, and 0.09 to − 0.03 where half a missing FA exists. The signal amplitude reduction ratios are 1.0% and 5.0%, respectively.

**Figure 8:**
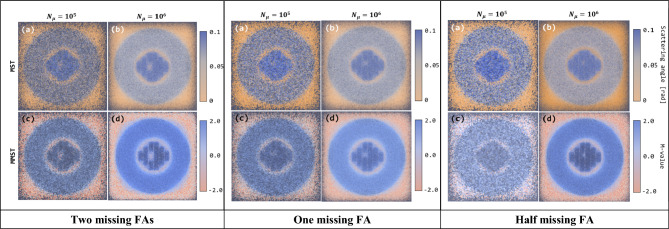
Reconstructed muon tomographic images for SNF assemblies, concrete overpacks, and airgap in the DSC when two, one, and one-half FAs are missing (from left to right). For each case, four cross-sectional images are presented that were reconstructed using the MST and MMST imaging algorithms with 10^5^ and 10^6^ muon samples, respectively. The overall image resolution is improved by either increasing the number of muon samples from 10^5^ to 10^6^ (left and right columns) or replacing the MST algorithm with the MMST algorithm (upper and lower rows).

The reconstructed muon tomographic images of the DSC with 0, 1/2, 1, and 2 missing FAs are shown in Fig. 8. In each case, four cross-sectional images are presented, which were reconstructed using the MST and MMST imaging algorithms with 10^5^ and 10^6^ muon samples, respectively. The overall image resolution is improved by increasing the number of muon samples (see and compare left and right columns in Fig. 8) and replacing scattering angles with *M-values* (see and compare upper and lower rows in Fig. 8). The best resolution can be achieved by using the MMST imaging algorithm with 10^6^ muon samples: one-half, one, and two missing FAs were successfully visually identified and located as shown in Fig. 8.

**Figure 9:**
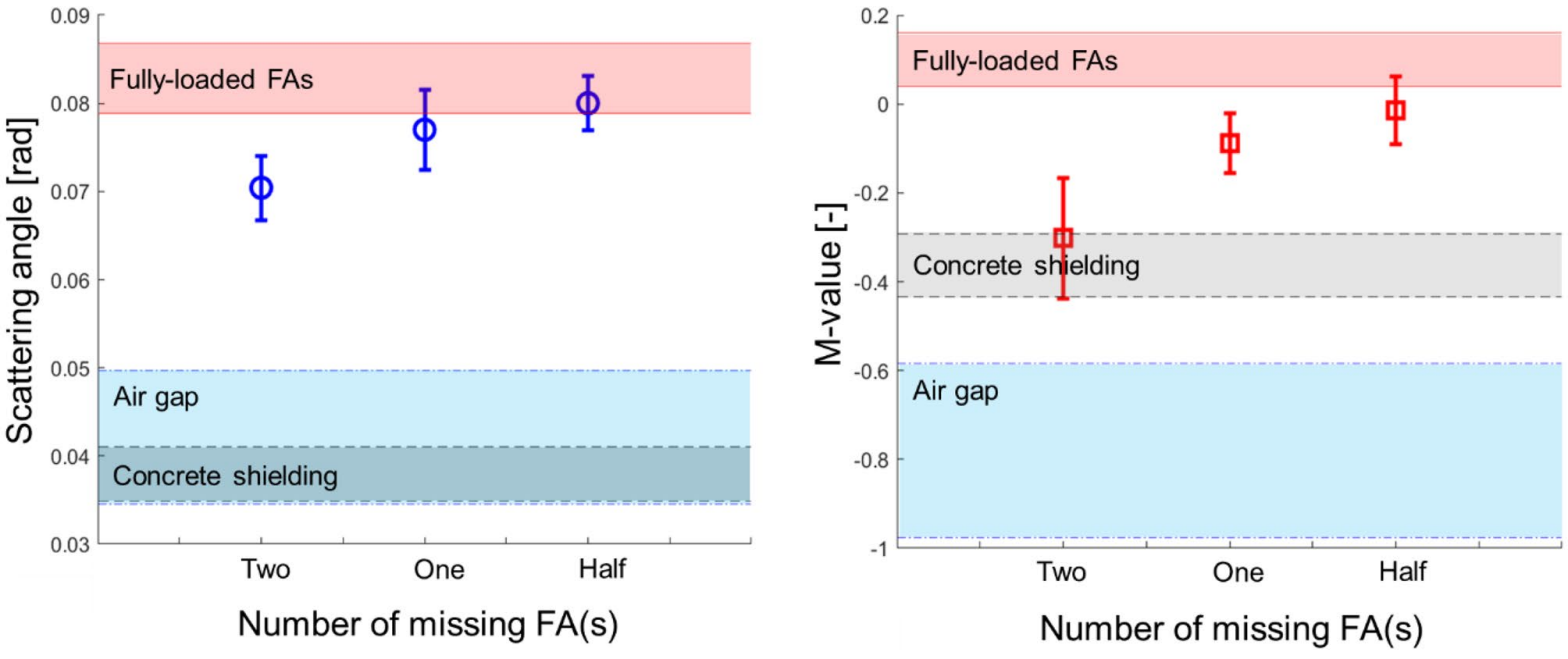
Visualized signal analyses in MST (left) and MMST (right) which present scattering angle and *M-value* signal amplitude distributions for fully loaded FAs (red), concrete shielding (gray), and airgap (blue) when one-half, one, and two FAs are missing. Signal stabilization, enhanced capability to distinguish various materials, and find missing FAs are observed. Error bars represent 1σ.

The benefits of using MMST algorithm—which offers distinctive signal amplitudes for different materials and an improved capability to identify and locate missing FAs—are summarized in Fig. 9. The ranges of scattering angle and *M-value* signal amplitudes for various structural materials in the DSC are presented in different colors in Fig. 9. *M-value* signals for all materials are clearly separated in MMST signal analysis, whereas scattering angle signals for the airgap and concrete are overlapped, which means two materials are indistinguishable in MST signal analysis. The averaged signal amplitudes of scattering angles and *M-values* for 1/2, 1, and 2 missing FAs’ are plotted with 1σ error bars. The scattering angle signals are overlapped with the fully loaded FAs signal area when one and two FAs are missing with less than 60% and 30% CLs. On the other hand, the *M-value* signals do not overlap with the fully loaded FAs signal area when one FA is missing (> 99% CL), and one-half FA can be found with an 80% CL.

## Conclusion

This work shows that the measurements of cosmic-ray muon energy using MST can improve the image resolution and enhance our ability to find and locate missing FAs in DSCs. To mathematically encode muon energy information to the existing MST imaging algorithm, a new algorithm, MMST, was developed. To demonstrate the benefits of MMST in the application for monitoring SNF, a commercial SNF dry cask canister that stores up to 24 PWR FAs was designed in a GEANT4 environment. The array of FAs is surrounded by an annular concrete shielding. Additionally, muon trackers and a Cherenkov spectrometry were also modeled using GEANT4 to simulate muon interactions with DSC materials and measure muon scattering angles and energy as shown in Fig. 6. To test the functionality of the MMST algorithm, four scenarios—with fully loaded, two, one, and one-half FAs missing—were considered and analyzed using two approaches, signal and image analyses. The results show three notable technical improvements enabled by the use of MMST: (1) a signal stabilization or reduced signal fluctuation, (2) enhanced capability to differentiate various DSC structural materials, and (3) statistically increased precision to identify and locate missing FAs. The reconstructed image quality is improved which enables to find the position and quantity of missing FAs in DSC investigations. The rates of correctly identifying missing FAs were improved from 79 to 98% and 51% to 88% by replacing scattering angles with *M-values* when one FA and one-half FA is missing, respectively. These improvements also can be visually observed in the reconstructed images (Fig. 9).

The advancement in the MST technique for DSC verification is expected to resolve long-standing problems in international nuclear safeguards and nuclear material accountancy^54^. With a traditional MST technique, approximately 90 days of measurement time is required to survey the entire DSC^17^. By utilizing muon energy information in the MST imaging algorithm, we found that the required measurement time is significantly reduced, and comparable results were acquired by the smaller number of muon samples to survey missing FAs in the DSC using MMST instead of MST. Given the current absence of fully licensed NDE techniques for monitoring SNF in DSCs, the MMST imaging algorithm emerges as a highly promising solution. Nevertheless, comprehensive field testing is required for it to attain licensure from authorized agencies like the Nuclear Regulatory Commission (NRC). Future investigations will focus on a sensitivity study of MMST, aimed at detecting missing fuel pins and monitoring material failures such as cracks, imperfections, and swelling in structural materials and fuel pins. Enhancing the resolution of MMST images necessitates: (1) improved muon momentum and position measurement capabilities, (2) enhanced particle tracking algorithms, and (3) increased muon flux. Enhancements in radiation detection instrumentation are expected to reduce statistical measurement uncertainties. Moreover, recent research demonstrates the feasibility of estimating the most probable muon trajectory within the target object using Bayesian theory-based algorithms^55^. While increased muon flux can be achieved through adaptation of a muon beam accelerator facility^56,57^ deployment in field conditions remains impractical.

## Data Availability

The datasets generated and/or analyzed during the current study are available from the corresponding author (JungHyun Bae) upon request.

## References

[1] EIA. U.S. Energy Information Administration [Online] (2022). Available: https://www.eia.gov/energyexplained/nuclear/the-nuclear-fuel-cycle.php [Accessed September 2023].

[2] Nechaev, A., Onufriev, V., & Thomas, K. T. Long-term storage and disposal of spent fuel.

[3] U.S. NRC. Dry Cask Storage. 2023. [Online]. Available: https://www.nrc.gov/waste/spent-fuel-storage/dry-cask-storage.html. [Accessed September 2023].

[4] El-Samrah, M., Zamora, M. A., Novog, D. & Chidiac, S. Radiation shielding properties of modified concrete mixes and their sustainability in dry storage cask. *Prog. Nuclear Energy* **148**, 1 (2022). 10.1016/j.pnucene.2022.104195

[5] Gao, Y., McFerran, N. J., Enqvist, A., Tulenko, J. E. & Baciak, J. E. Dry cask radiation shielding validation and estimation of cask surface dose rate with MAVRIC during long-term storage. *Ann. Nuclear Energy* **140**, 1 (2020). 10.1016/j.anucene.2019.107145

[6] Bae, J., Bean, R. & Abboud, R. CFD analysis of a dry storage cask with advanced spent nuclear fuel cask additives. *Ann. Nuclear Energy* **145**, 107 (2020). 10.1016/j.anucene.2020.107610

[7] Bolotina, I., Bulavinov, A., Lider, A., Sednev, D. & Shtaynbreher, A. Ultrasonic inspection of spent nuclear fuel casks. *IOP Conf. Series: Mater. Sci. Eng.* **81**, 1 (2015).

[8] Salchak, Y. *et al.* Dry storage casks monitoring by means of ultrasonic tomography. *Phys. Proc.* **70**, 484–487 (2015). 10.1016/j.phpro.2015.08.291

[9] Liu, X. & Lee, H. K. A simulation study of the spent nuclear fuel cask condition evaluation using high energy X-ray computed tomography. *NDT E Int.* **80**, 58–64 (2016). 10.1016/j.ndteint.2016.02.008

[10] Greulich, C., Hughes, C., Gao, Y., Enqvist, A. & Baciak, J. High energy neutron transmission analysis of dry cask storage. *Nucl. Instrum. Methods Phys. Res. Sect. A* **874**, 5–11 (2017). 10.1016/j.nima.2017.08.014

[11] Liu, Z., Fang, M., George, J., Meng, L.-J. & Fulvio, A. D. Neutron tomography of spent fuel casks. *J. Signal Process. Syst.* **94**, 399–409 (2022). 10.1007/s11265-021-01706-7

[12] Y. Ham, S. Sitarman and P. Kerr, "Verification of Spent Fuel Inside Dry Storage Casks using Fast Neutrons," in *ESARDA Symposium*, 2019.

[13] Miyadera, H. & Morris, C. L. Muon scattering tomography: Review. *Appl. Opt.* **61**(6), 154–161 (2022). 10.1364/AO.445806 35201040

[14] Schultz, L. *et al.* Image reconstruction and material Z discrimination via cosmic ray muon radiography. *Nucl. Instrum. Methods Phys. Res. Sect. A* **519**, 687–694 (2004). 10.1016/j.nima.2003.11.035

[15] Borozdin, K. *et al.* Radiographic imaging with cosmic-ray muons. *Nature* **422**, 277 (2003). 12646911 10.1038/422277a

[16] Poulson, D. *et al.* Cosmic ray muon computed tomography of spent nuclear fuel in dry storage casks. *Nuclear Instrum. Methods Phys. Res. Sect. A* **842**, 48–53 (2017). 10.1016/j.nima.2016.10.040

[17] Durham, J. *et al.* erification of spent nuclear fuel in sealed dry storage casks via measurements of cosmic-ray muon scattering. *Phys. Rev. Appl.* **9**, 1 (2018). 10.1103/PhysRevApplied.9.044013

[18] Chatzidakis, S., Choi, C. & Tsoukalas, L. Interaction of cosmic ray muons with spent nuclear fuel dry casks and determination of lower detection limit. *Nuclear Instrum. Methods Phys. Res. Sect. A* **828**, 37–45 (2016). 10.1016/j.nima.2016.03.084

[19] Jonkmans, G., Anghel, V., Jewett, C. & Thompson, M. Nuclear waste imaging and spent fuel verification by muon tomography. *Ann. Nuclear Energy* **53**, 267–273 (2013). 10.1016/j.anucene.2012.09.011

[20] Park, C. *et al.* Design and characterization of a muon tomography system for spent nuclear fuel monitoring. *Nuclear Eng. Technol.* **54**, 601–607 (2022). 10.1016/j.net.2021.08.029

[21] Chatzidakis, S., Choi, C. & Tsoukalas, L. Investigation of imaging spent nuclear fuel dry casks using cosmic ray muons. *Trans. Am. Nucl. Soc.* **114**, 1 (2016).

[22] Chatzidakis, S., Hausladen, P., Croft, S., Chapman, J., Jarrell, J., Scaglione, J., Choi, C., & Tsoukalas, L. Classification and imaging of spent nuclear fuel dry casks using cosmic ray muons. In *Nucl. Plant Instrum. Control Hum. Mach. Interface Technol,* pp. 237–245 (2017).

[23] Li, Y. *et al.* Muon scattering tomography of spent fuel dry storage casks. *J. Instrum.* **14**, 2001 (2019). 10.1088/1748-0221/14/12/C12001

[24] Bae, J. & Chatzidakis, S. Monitoring spent nuclear fuel in a dry cask using momentum integrated muon scattering tomography. *Trans. Am. Nuclear Soc.* **127**, 828–832 (2022).

[25] Bertoni, R. *et al.* The design and commissioning of the MICE upstream time-of-flight system. *Nucl. Instrum. Methods Phys. Res. Sect. A* **615**, 14–26 (2010). 10.1016/j.nima.2009.12.065

[26] Vallance, C. *et al.* Fast sensors for time-of-flight imaging applications. *Phys. Chem. Chem. Phys.* **16**, 383–395 (2014). 24002354 10.1039/C3CP53183J

[27] Abratenko, P. *et al.* Determination of muon momentum in the MicroBooNE LArTPC using an improved model of multiple Coulomb scattering. *J. Instrum.* **12**, 1 (2017). 10.1088/1748-0221/12/10/P10010

[28] Topuz, A., Kiisk, M., Giammaco, A. & Mägi, M. Effect of passive metallic layers on muon energy estimation by means of deflection angle for muon scattering tomography: A comparative study based on GEANT4 simulations. *J. Instrum.* **17**, 2008 (2022). 10.1088/1748-0221/17/02/C02008

[29] Wang, L. *et al.* Cosmic ray mass independent energy reconstruction method using Cherenkov light and muon content in LHAASO. *Phys. Rev. D* **107**, 4 (2023).

[30] Martins, E. P. & de Souza, V. On the detection of direct Cherenkov light from ultrahigh-energy cosmic rays. *Astropart. Phys.* **141**, 102 (2022).

[31] Bae, J. & Chatzidakis, S. Fieldable muon spectrometer using multi-layer pressurized gas Cherenkov radiators and its applications. *Sci. Rep.* **12**, 1 (2022). 35169208 10.1038/s41598-022-06510-2 PMC8847616

[32] Bae, J. & Chatzidakis, S. Fieldable muon momentum measurement using coupled pressurized gaseous cherenkov detectors. *Trans. Am. Nuclear Soc.* **125**, 400–403 (2022).

[33] Bae, J. & Chatzidakis, S. Development of compact muon spectrometer using multiple pressurized gas Cherenkov radiators. *Res. Phys.* **39**, 105 (2022). 10.1038/s41598-022-06510-2 PMC8847616 35169208

[34] Schultz, L. J. *et al.* Image reconstruction and material Z discrimination via cosmic ray muon radiography. *Nuclear Instrum. Methods Phys. Res. Sect. A* **519**, 687–694 (2004). 10.1016/j.nima.2003.11.035

[35] Schultz, L. J. *et al.* Statistical reconstruction for cosmic ray muon tomography. *IEEE Trans. Image Process.* **16**, 1985–1993 (2007). 17688203 10.1109/TIP.2007.901239

[36] Allison, J. *et al.* Geant4 developments and applications. *IEEE Trans. Nuclear Sci.* **53**, 270–278 (2006). 10.1109/TNS.2006.869826

[37] Agostinelli, S. *et al.* GEANT4—a simulation toolkit. *Nucl. Instrum. Methods Phys. Res. Sect. A* **506**, 250–303 (2003). 10.1016/S0168-9002(03)01368-8

[38] Workman, R. L. *et al.* The review of particle physics. *Prog. Theor. Exp. Phys.* **8**, 2022 (2022).

[39] NIST. CODATA Value: muon-electron mass ratio. *The NIST Reference on Constants, Units, and Uncertainty* (2019).

[40] Bae, J. & Chatzidakis, S. A new semi-empirical model for cosmic ray muon flux estimation. *Prog. Theor. Exp. Phys.* **1**, 1 (2022).

[41] Grieder, P. K. F. Cosmic Rays at Earth, Elsevier Science (2001).

[42] Bethe, H. A. Molière’s theory of multiple scattering. *Phys. Rev.* **89**, 1256–1266 (1953). 10.1103/PhysRev.89.1256

[43] Highland, V. L. Some practical remarks on multiple scattering. *Nuclear Instrum. Methods* **129**, 497–499 (1975). 10.1016/0029-554X(75)90743-0

[44] Particle Data Group. Atomic and Nuclear Properties of Materials for more than 350 materials (2020). [Online]. Available: https://pdg.lbl.gov/2020/AtomicNuclearProperties/index.html.

[45] Tsai, Y. Pair production and bremsstrahlung of charged leptons. *Rev. Mod. Phys.* **16**, 815–851 (1974). 10.1103/RevModPhys.46.815

[46] Chatzidakis, S., Liu, Z., Hayward, J. P. & Scaglionw, J. M. A generalized muon trajectory estimation algorithm with energy loss for application to muon tomography. *J. Appl. Phys.* **123**, 12 (2018). 10.1063/1.5024671

[47] Benettoni, M. *et al.* Noise reduction in muon tomography for detecting high density objects. *J. Instrum.* **8**, 1 (2013). 10.1088/1748-0221/8/12/P12007

[48] Motoki, M. *et al.* Precise measurements of atmospheric muon fluxes with the BESS spectrometer. *Astropart. Phys.* **19**, 113–126 (2003). 10.1016/S0927-6505(02)00195-0

[49] Haino, S. *et al.* Measurements of primary and atmospheric cosmic-ray spectra with the BESS-TeV spectrometer. *Phys. Lett. B* **594**, 35–46 (2004). 10.1016/j.physletb.2004.05.019

[50] Schwerdt, C. Zenith angle dependence. Wissenschaftliche Koordinatorin Cosmic-Projekte (2018).

[51] EPRI. Industry spent fuel handbook. Technical Report 1021048 (2010).

[52] U.S NRC. Standards for protection against radiation, Regulations 10 CFR Part 20 (2021).

[53] Bae, J., Montgomery, R. & Chatzidakis, S. Image reconstruction algorithm for momentum dependent muon scattering tomography. *Nucl. Eng. Technol.* **1**, 1 (2023).

[54] Bae, J., Montgomery, R. & Chatzidakis, S. Nuclear material accountancy using momentum-informed muon scattering tomography. *Ann. Nuclear Energy* **197**, 110240 (2024). 10.1016/j.anucene.2023.110240

[55] Ughade, R., Bae, J. & Chatzidakis, S. Performance evaluation of cosmic ray muon trajectory estimation algorithms. *AIP Adv.* **13**, 125301 (2023). 10.1063/5.0174796

[56] Chen, C.-Y., Pospelov, M. & Zhong, Y.-M. Muon beam experiments to probe the dark sector. *Phys. Rev. D* **95**(11), 1 (2017). 10.1103/PhysRevD.95.115005

[57] Cook, S. *et al.* Delivering the world’s most intense muon beam. *Phys. Rev. Accel Beams* **20**(3), 030101 (2017). 10.1103/PhysRevAccelBeams.20.030101

