# Author Correction: Momentum informed muon scattering tomography for monitoring spent nuclear fuels in dry storage cask

**DOI:** 10.1038/s41598-024-60641-2

**Published:** 2024-05-17

**Authors:** JungHyun Bae, Rose Montgomery, Stylianos Chatzidakis

**Affiliations:** 1 Oak Ridge National Laboratory, Oak Ridge, TN 37830 USA; 2 School of Nuclear Engineering, Purdue University, West Lafayette, IN 47907 USA

Correction to: *Scientific Reports* 10.1038/s41598-024-57105-y, published online 20 March 2024

The original Article has been corrected.

“© The Author(s) 2024”

now reads:

“© UT-Battelle, LLC 2024”

The original Article has been corrected.

